# Development and Validation of a Dynamic Nomogram to Predict the Risk of Neonatal White Matter Damage

**DOI:** 10.3389/fnhum.2020.584236

**Published:** 2021-02-23

**Authors:** Wenjun Cao, Chenghan Luo, Mengyuan Lei, Min Shen, Wenqian Ding, Mengmeng Wang, Min Song, Jian Ge, Qian Zhang

**Affiliations:** Neonatal Intensive Care Unit, The First Affiliated Hospital of Zhengzhou University, Zhengzhou, China

**Keywords:** neonatal, white matter damage, nomogram, prediction model, perinatal, web-based calculator

## Abstract

**Purpose:**

White matter damage (WMD) was defined as the appearance of rough and uneven echo enhancement in the white matter around the ventricle. The aim of this study was to develop and validate a risk prediction model for neonatal WMD.

**Materials and Methods:**

We collected data for 1,733 infants hospitalized at the Department of Neonatology at The First Affiliated Hospital of Zhengzhou University from 2017 to 2020. Infants were randomly assigned to training (*n* = 1,216) or validation (*n* = 517) cohorts at a ratio of 7:3. Multivariate logistic regression and least absolute shrinkage and selection operator (LASSO) regression analyses were used to establish a risk prediction model and web-based risk calculator based on the training cohort data. The predictive accuracy of the model was verified in the validation cohort.

**Results:**

We identified four variables as independent risk factors for brain WMD in neonates by multivariate logistic regression and LASSO analysis, including gestational age, fetal distress, prelabor rupture of membranes, and use of corticosteroids. These were used to establish a risk prediction nomogram and web-based calculator (https://caowenjun.shinyapps.io/dynnomapp/). The C-index of the training and validation sets was 0.898 (95% confidence interval: 0.8745–0.9215) and 0.887 (95% confidence interval: 0.8478–0.9262), respectively. Decision tree analysis showed that the model was highly effective in the threshold range of 1–61%. The sensitivity and specificity of the model were 82.5 and 81.7%, respectively, and the cutoff value was 0.099.

**Conclusion:**

This is the first study describing the use of a nomogram and web-based calculator to predict the risk of WMD in neonates. The web-based calculator increases the applicability of the predictive model and is a convenient tool for doctors at primary hospitals and outpatient clinics, family doctors, and even parents to identify high-risk births early on and implementing appropriate interventions while avoiding excessive treatment of low-risk patients.

## Introduction

The survival of newborns with serious disease has significantly improved in recent years as a result of advances in neonatal intensive care (Cheng et al., 2015); however, the occurrence of sequelae in surviving children that can affect their physical and mental development has also increased, placing a burden on their families and society. The sequelae include intracranial hemorrhage, brain white matter damage (WMD), and hypoxic ischemia (Gale et al., 2018). WMD is the most common type of perinatal brain damage and the main cause of permanent motor and cognitive impairment (Keunen et al., 2015; Serdar et al., 2016).

White matter damage detected by ultrasonography usually presents in four stages: (1) echogenicity enhancement stage; (2) relatively normal period; (3) cyst formation stage; and (4) disappearance of the cysts (Resch et al., 2006). A cystic change in the cranial ultrasound reflects periventricular white matter softening, which is associated with an increased risk of neurologic disorders such as cerebral palsy (Graham et al., 1987; Resić et al., 2008; Sarkar et al., 2018). During echogenicity enhancement, the periventricular echodensity (PVE) 1 level occurs when the echogenicity of the white matter around the ventricle is enhanced to be as bright as choroid plexus. The echo intensity of periventricular white matter was higher than that of the choroid plexus, PVE2 (Resch et al., 2006). It was previously assumed that PVE in premature infants had little clinical significance as it was a common finding (Grant et al., 1983; DiPietro et al., 1986). However, more recent studies have shown that the strong echo around the ventricle does not fade within a certain period, which can also reflect the severity of white matter injury (Wang et al., 2017). Besides, the duration of periventricular echodensities, rather than grade, is significantly associated with abnormal neurodevelopmental outcomes independent of gestational age (GA) (Resch et al., 2006). When no abnormality or only slight echogenicity is observed in the cranial ultrasound scan for a period of less than 1 week, the enhanced echogenicity may be caused by venous congestion (De Vries et al., 1988; de Vries et al., 1992) or simple immaturity (Resch et al., 2006), which has little impact on the long-term prognosis of neonates. However, if the echogenicity around the ventricle is abnormal for more than 7 days, there is an increased risk of long-term neurologic dysfunction such as abnormal movement, mental retardation, and sensory loss (de Vries et al., 1992; Ringelberg and van de Bor, 1993; Kutschera et al., 2006). As such, its prognosis cannot be ignored (Resch et al., 2006).

The early clinical manifestations of WMD vary, and diagnosis depends on imaging examination. Most doctors perform cranial ultrasound screening for premature infants, especially those born earlier than 32 weeks of gestation (Volpe, 2003). In clinical practice, 34–39% of WMD occurs in the near term (Wu et al., 2014; Jensen and Holmer, 2018), although most studies have not differentiated between full- and near-term cases (Berger et al., 1997; Wu et al., 2014; Jensen and Holmer, 2018). The lack of cranial ultrasound screening at full or near term may be the reason for which, in some children, the cause of developmental retardation and cerebral palsy is unknown. There is a need for effective, convenient, and inexpensive tools to assess the risk of WMD for all newborns.

Nomograms are predictive models that can be used to determine disease risk and survival outcome and have been widely used in medicine (e.g., in cancer) to guide clinical treatment (Liu S.L. et al., 2020, Liu Q. et al., 2020; Zhou et al., 2020). There is currently no nomogram for predicting the risk of WMD. Therefore, in this study, we established a nomogram for WMD based on variables that were identified as being relevant before and at birth (Gilles and Leviton, 2020). The nomogram does not require any invasive or non-invasive testing and can be applied immediately after birth to assess the risk of WMD. According to the results, early brain function monitoring and nutritional nerve treatment to prevent the occurrence of cerebral palsy and other neurological sequelae should be carried out for high-risk infants, early intervention should be conducted, and overdiagnosis and treatment should be avoided for low-risk infants, so as to promote the development of precision medicine.

## Materials and Methods

### Patients

This study enrolled newborns who were admitted to the neonatal intensive care unit of The First Affiliated Hospital of Zhengzhou University from June 2017 to June 2020 and survived until discharge. Newborns with congenital metabolic defects or malformations, chromosomal abnormalities, death or abandonment of medical treatment by family members, who were not screened by cephalic ultrasound because of short hospital stay or the family’s disapproval, and whose general information was incomplete were excluded.

Data collected from the medical records of the mother and infant included sex, cesarean delivery, GA, birth weight (BW), multiple pregnancies, Apgar 1- and 5-min scores, placental abnormalities (including placenta previa, implantation, abruption, morphogenesis), abnormal umbilical cord, amniotic fluid anomalies, hypertension, gestational diabetes mellitus, treatments during pregnancy (e.g., progesterone), prelabor rupture of membranes (PROM) (duration > 12 h), maternal age, embryo transfer, adverse pregnancy history, cardiac dysfunction, and use of corticosteroids (CS). Generally, CS is considered to be routinely used on pregnant women at 24–33 + 6 weeks of gestation as well as at risk of preterm birth at 7 days, fetal growth restriction, and weight or abdominal circumference below the 10th percentile of the corresponding GA estimated by prenatal fetal ultrasound (Lausman and Kingdom, 2013). Hypothyroidism during pregnancy was defined as a serum thyroid-stimulating hormone level exceeding the upper limit of the reference value range and serum free thyroxine 4 level lower than the reference range during pregnancy (Alexander et al., 2017). Thyroid function testing was performed for all pregnant women. Intrahepatic cholestasis of pregnancy (ICP) was defined as fasting blood total bile acid level ≥ 10 mol/l (Hardikar et al., 2009). Serum bile acid levels were measured in pregnant women with itchy skin, jaundice, and elevated liver enzyme and bilirubin levels. Fetal distress (FD) was defined as prenatal distress symptoms, fetal heart rate ≥ 160 or ≤ 120 times/min for a duration ≥ 1 min, late deceleration (Moors et al., 2020). All pregnant women have cardiotocography (CTG) examination before delivery.

### Neonatal Ultrasound Examination

During the hospitalization, ultrasound evaluation was performed by experienced pediatric radiologists in the 1st week of birth (postnatal days 3–5) and 2nd week of birth (postnatal days 10–14) with the informed consent of the family.

White matter damage was diagnosed by cranial ultrasound using an Arietta 70 color Doppler ultrasound machine (Hitachi, Tokyo, Japan) with the convex array probe frequency set at 4.0–8.0 MHz. We scanned through the anterior fontanelle in the coronal and sagittal planes in order to sequentially observe the frontal lobe, anterior horn of the lateral ventricle, third ventricle, central part of the lateral ventricle to the posterior horn, and occipital lobe. The intensity, size, distribution, shape, bilaterality, and other characteristics of the strong echo region of the white matter around the ventricle were recorded. If the echo intensity around the ventricle was similar to that of the choroid plexus and did not develop into a cyst for more than 7 days, it was considered to be WMD group. If the white matter had normal echo intensity or if the echo enhancement lasted less than 7 days, it was considered as non-WMD group. The study was approved by the Ethics Committee of The First Affiliated Hospital of Zhengzhou University.

### Statistical Analysis

SPSS v25.0 software was used to compare the perinatal conditions of infants in the training and verification cohorts. For quantitative data, we performed normality testing (Shapiro–Wilk test); GA, BW, Apgar 1- and 5-min scores, and maternal age were found to be non-normally distributed, and these data are presented as median (upper quartile, lower quartile). The Wilcoxon and χ^2^ tests were used to compare qualitative data between cohorts. Differences were considered statistically significant at *P* < 0.05.

### Generation of the Nomogram

We used R v3.6.3 software^1^ to build the risk prediction model. The least absolute shrinkage and selection operator (LASSO) method, which is suitable for high-dimensional data compression, was used to select newborn risk factors with the highest predictive value (Boulesteix et al., 2017; Sauerbrei et al., 2020) (i.e., with non-zero coefficients) (Kidd et al., 2018). The factors screened by LASSO regression were included in the multivariate logistic regression analysis to establish a prediction model. Differences with *P* < 0.05 were considered statistically significant. All potential predictors identified in previous cohort studies (Iasonos et al., 2008; Balachandran et al., 2015) were included in the neonatal WMD risk prediction model. The performance of the nomogram was evaluated by determining the concordance (C-)index, sensitivity, specificity, receiver operating characteristic (ROC) curve, and calibration (calibration chart). Decision curves were plotted to assess the clinical utility of the nomogram (Wang et al., 2018). Additionally, we used the “shiny” and “DynNom” packages in R software to generate a web-based calculator (using training queues) to dynamically predict the WMD risk^2^.

## Results

### Baseline Characteristics of Infants

Ultimately, a total of 1,733 cases were collected. A total of 157 (9.06%) infants were in the WMD group, and 1,576 (90.94%) were in the non-WMD group. These infants were randomly assigned to a training cohort (*n* = 1,216) or verification cohort (*n* = 517) at a ratio of 7:3. Baseline characteristics of the cohorts are shown in Table 1. The training cohort (*n* = 1,216) was used to build predictive models and predict risk stratification, and the prediction model was validated using the verification group.

**TABLE 1:** Baseline characteristics of cohort.

	**Total**	**Traning cohort**	**Validation cohort**	**P/Z**
WMD(%)	157(9.06)	114(9.38)	43(8.32)	0.483
**Birth characteristic**				
Male (%)	923(53.26)	667(54.85)	256(49.52)	0.042
Cesarean delivery (%)	1,163(67.11)	810(66.61)	353(68.28)	0.499
Multiple pregnancy (%)	240(13.85)	182(14.97)	58(11.22)	0.039
GA (weeks) [median (IQR)]	36.86 (34.29,39.14)	36.86 (34.29,39.14)	36.86 (34.43,39.29)	0.320
BW (g) [median (IQR)]	2,700 (2,050, 3,300)	2,700 (2,000, 3,300)	2,750 (2,100, 3,350)	0.313
Apgar score, 1 min [median (IQR)]	9(8,10)	9(8,10)	10(8,10)	0.532
Apgar score, 5 min [median (IQR)]	10(9,10)	10(9,10)	10(10,10)	0.464
Abnormal umbilical cord (%)	465(26.83)	324(26.64)	141(27.27)	0.787
Placental abnormality (%)	304(17.54)	210(17.27)	94(18.18)	0.648
Amniotic fluid anomaly (%)	508(29.31)	350(28.78)	158(30.56)	0.457
**Maternal characteristics**				
Maternal age (years) [median (IQR)]	30(27,33)	30(28,33)	29(27,32)	0.034
GDM (%)	300(17.31)	217(17.85)	83(16.05)	0.367
Hypothyroidism with pregnancy (%)	94(5.42)	66(5.43)	28(5.42)	0.992
Hypertension (%)	295(17.02)	207(17.02)	88(17.02)	0.999
ICP (%)	27(1.56)	17(1.40)	10(1.93)	0.410
Cardiac dysfunction (%)	13(0.75)	5(0.41)	8(1.55)	0.027
History of abnormal pregnancy (%)	178(10.27)	133(10.94)	45(8.70)	0.161
Maternal disease (%)	326(18.81)	234(19.24)	92(17.79)	0.48
**Pregnancy-related factors**				
Embryo transfer (%)	324(18.70)	243(19.98)	81(15.67)	0.035
Fetal distress (%)	203(11.71)	140(11.51)	63(12.19)	0.69
IUGR (%)	67(3.87)	44(3.62)	23(4.45)	0.412
Use of CS (%)	193(11.14)	132(10.86)	61(11.80)	0.568
Pregnancy treatment (%)	247(14.25)	171(14.06)	76(14.70)	0.728
PROM (%)	416(24.00)	302(24.84)	114(22.05)	0.214

### Selection of Perinatal Characteristics

**FIGURE 1:**
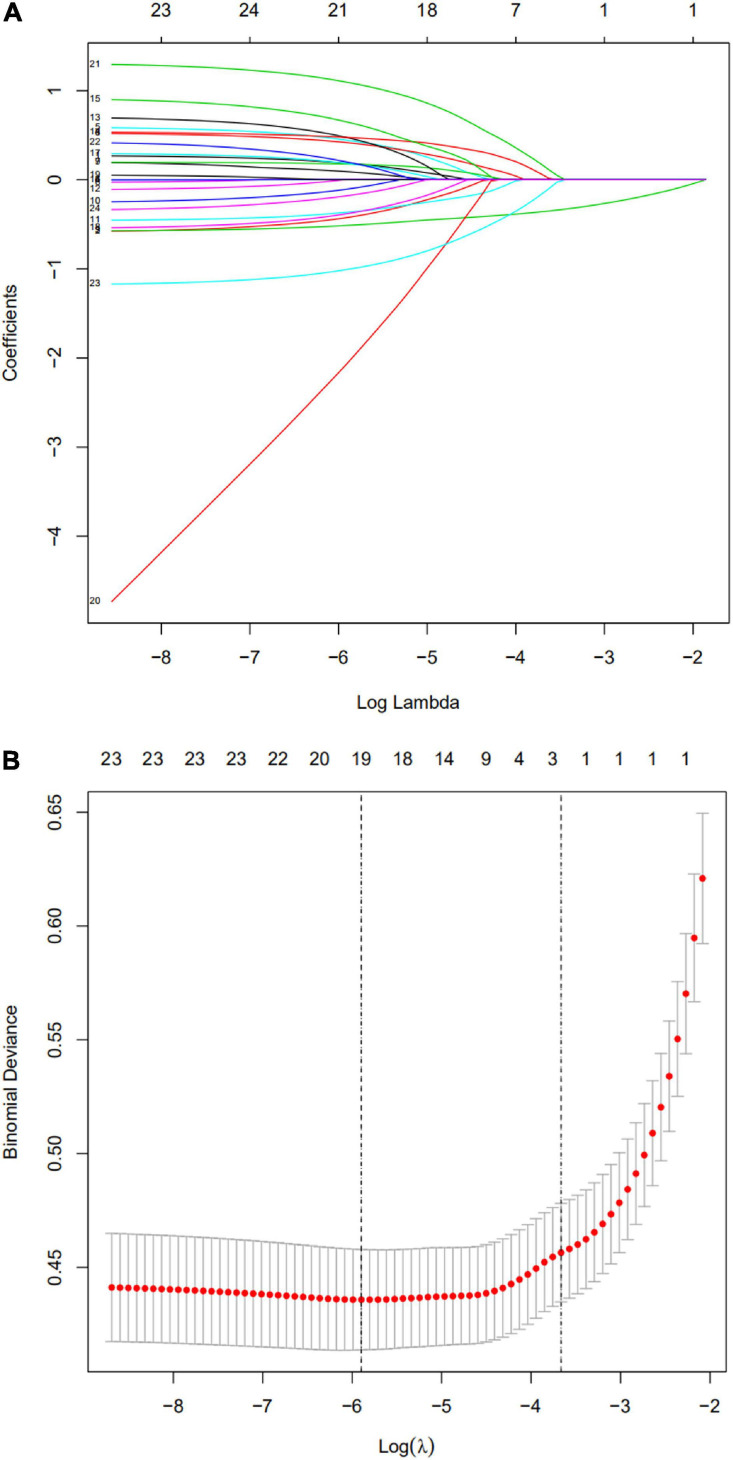
Selection of perinatal factors using the least absolute shrinkage and selection operator (LASSO) binary logistic regression model. **(A)** The predictive factors were determined by cable regression method. For the optimal lambda, five features with non-zero coefficients were selected. **(B)** Adjusting the penalty coefficient in the LASSO model using cross validation and minimum criteria. The vertical black line represents the optimal lambda (i.e., the model provides the best fit with the data). Therefore, the optimal λ was 0.0256, with log(λ) = –3.6652.

Based on the training cohort data, we used LASSO regression analysis to identify independent risk factors affecting WMD (Figures 1A,B). The coefficient, λ, decreases with an increase in the number of variables. When λ is optimal, the coefficient of excluded variables is compressed to 0, while the variable is not 0 in the selected model. The results of our analysis indicated that the optimal value of λ was 0.0256, with log(λ) = –3.6652. According to LASSO analysis results, the 24 perinatal correlation factors were reduced to five potential predictors (∼5:1 ratio). These characteristics include GA, BW, FD, PROM, and the use of CS (Table 2).

**TABLE 2:** Prediction factors for the brain WMD.

		**Prediction model**	
**Variable**	**B**	**OR (95% CI)**	**P**

GA	−0.52300	0.59 (0.5–0.69)	<0.001***
BW	0.00007	1.0001 (0.9994–1.0007)	0.837
PROM	0.73381	2.08 (1.28–3.4)	0.004**
Fetal distress	1.03410	2.81 (1.52–5.19)	0.001***
Use of CS	−1.12661	0.32 (0.16–0.65)	<0.001**

*p < 0.05; **p < 0.01; ***p < 0.001; BW, birth weight; CS, corticosteroids; GA, gestational age; OR, odds ratio; PROM, prelabor rupture of membranes; WMD, white matter damage.

### Development and Verification of Nomogram

**FIGURE 2:**
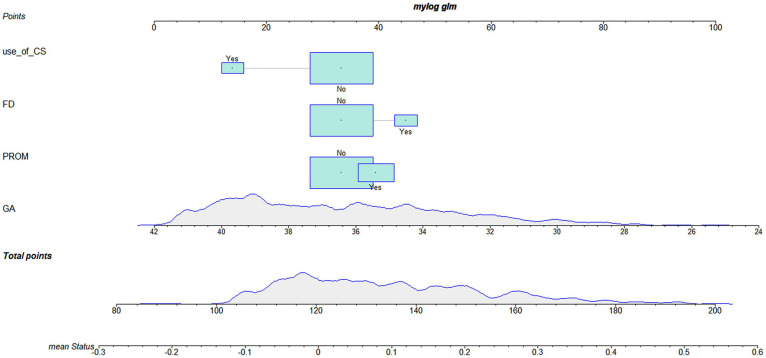
Nomogram for white matter damage (WMD).

**FIGURE 3:**
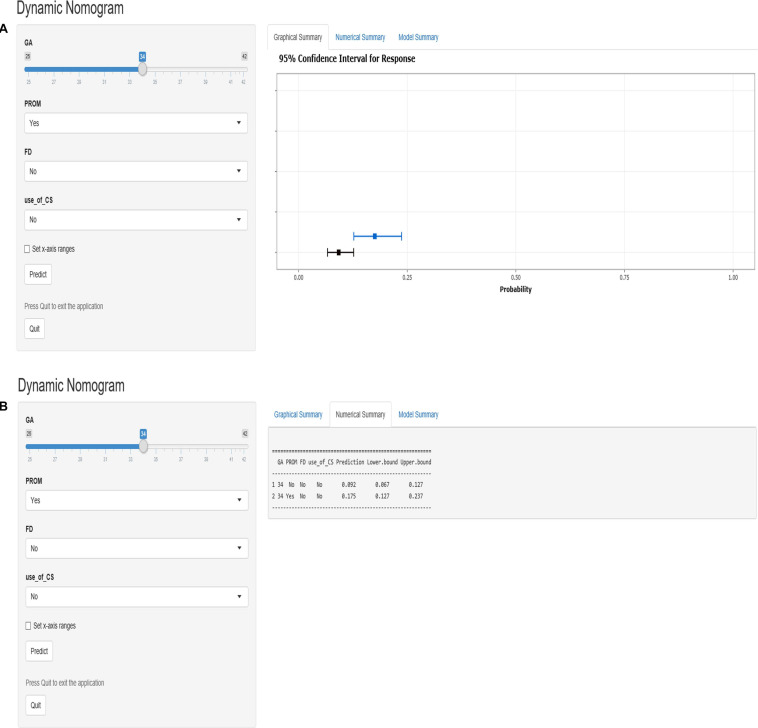
The dynamic nomogram of white matter damage (WMD). For example, if a newborn with a gestational age (GA) of 34 weeks and had a mother with no corticosteroids (CS) use during pregnancy and no prelabor rupture of membranes (PROM) or fetal distress (FD), the probability of WMD was determined to be 0.092 [95% confidence interval (CI): 0.067–0.127]. As this was lower than the cutoff value, the newborn was classified as belonging to the low-risk group. On the other hand, if a single newborn with a GA of 34 weeks and had a mother with no CS use during pregnancy and no FD, but had PROM, the probability of WMD was 0.175 (95% CI: 0.127–0.237). As this was higher than the cutoff value, this newborn belonged to the high-risk group that requires early screening, regular follow-up, and intervention when necessary **(A,B)**.

According to the logistic regression results, four variables with *P* < 0.05 were used to construct the risk prediction nomogram (Figure 2) and web-based calculator^3^ (Figures 3A,B).

For example, if a single newborn with a GA of 34 weeks and had a mother with no CS use during the pregnancy and no PROM or FD, the probability of WMD was determined to be 0.092 [95% confidence interval (CI): 0.067–0.127]; as this was lower than the cutoff value, the newborn was classified as belonging to the low-risk group. On the other hand, if a single newborn with a GA of 34 weeks and had a mother with no CS use during the pregnancy and no FD, but had PROM, the probability of WMD was 0.175 (95% CI: 0.127–0.237). As this was higher than the cutoff value, this newborn belonged to the high-risk group that requires early screening, regular follow-up, and intervention when necessary (Figures 3A,B).

**FIGURE 4:**
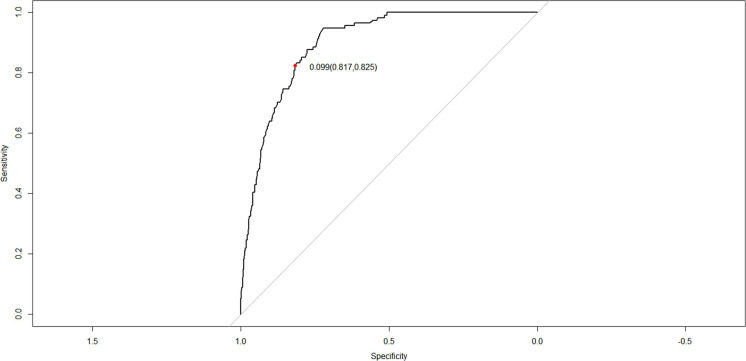
Receiver operating characteristic (ROC) curves of the nomogram for the prediction of white matter damage (WMD) in the training cohort.

The C-index for the nomogram in the training and verification cohorts was 0.898 (95% CI: 0.8745–0.9215) and 0.887 (95% CI: 0.8478–0.9262), respectively, indicating that the model had good discriminatory and predictive abilities. The ROC of the nomogram showed that the model had relatively high accuracy (Figure 4), with an area under the curve of 0.8979, sensitivity of 82.5%, specificity of 81.7%, and Youden index of 0.099.

### Clinical Utility of the Nomogram

**FIGURE 5:**
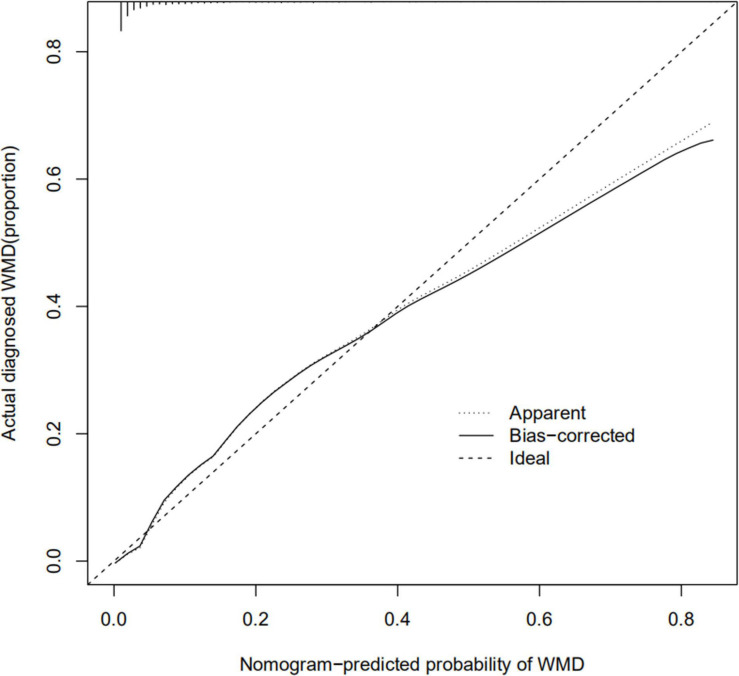
Calibration curve of nomogram for the prediction of white matter damage (WMD) in the training cohort.

**FIGURE 6:**
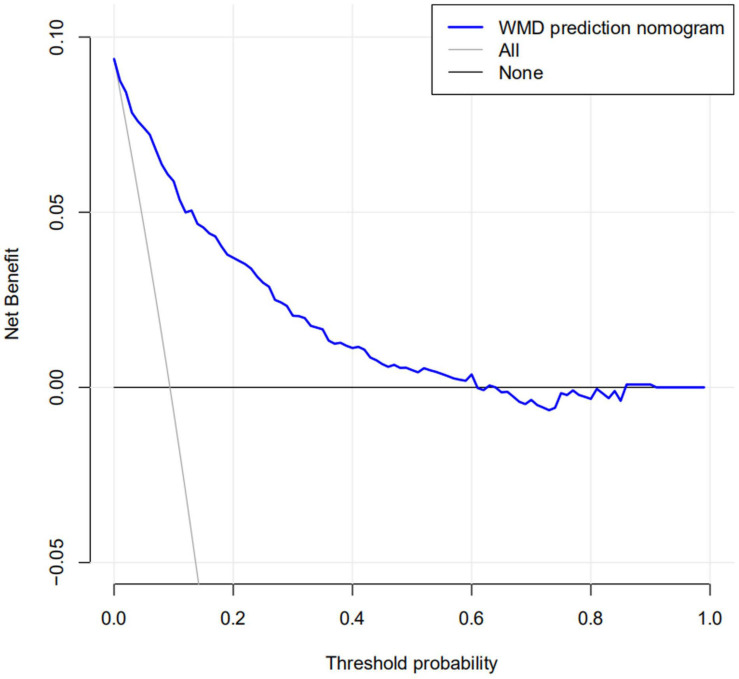
Nomogram decision curve for white matter injury in the training cohort.

The calibration curve for the nomogram predicting neonatal WMD risk indicated good consistency in this cohort (Figure 5). The decision curve analysis showed that by using patient or physician thresholds ranging from 1 to 61%, the nomogram could predict neonatal cerebral WMD risk with higher accuracy than intervention for all newborns and intervention for no newborns (Figure 6).

## Discussion

The goal of precision medicine is to develop highly targeted and personalized treatments and follow-up strategies for patients; this requires a highly accurate and convenient survival prediction model (Kent et al., 2018). A nomogram is a risk prediction model that can be applied to individual patients and is widely used for medical diagnosis and determination of clinical outcomes (Gold et al., 2009; Liang et al., 2015; Huang et al., 2016). Ours is the first study to use a nomogram to predict neonatal WMD risk based on four clinically relevant variables. The internal validation analysis revealed good discriminatory and calibration capabilities. We also developed a web-based calculator that allows clear visualization and dynamic prediction of WMD risk in neonates (Lin et al., 2020).

GA, PROM, FD, and use of CS were identified as independent risk factors for WMD. PROM and FD can increase the risk of WMD, while both higher GA and CS improve maturation and reduce the risk of WMD. Among them, GA has the greatest influence. Previous studies have shown that lower GA (especially < 32 weeks) is associated with an increased risk of WMD resulting from lack of maturation of blood vessels supplying the brain white matter and long and short perforating branches of the middle cerebral artery; almost no anastomoses in the cerebrovasculature; and absence of automatic adjustment thereof during development, which decreases systemic blood pressure and leads to passive-pressure cerebral circulation (Dammann et al., 2002; Nosarti et al., 2004; Leviton and Gressens, 2007; Rees et al., 2011; Mao et al., 2020). Oligodendrocyte precursor cells are the most abundant cell population in white matter during the preterm period and are particularly vulnerable to ischemia and inflammation because they lack antioxidant and other defense mechanisms (Back et al., 2001; Gibson and Clowry, 2003).

Our results suggest that FD may increase the risk of WMD. The brain is a highly metabolically active organ that is sensitive to hypoxia. In FD, the brain is in an anoxic state while the cerebrovascular system is in a state of pressure-passive circulation, with poor regulation of the response to injury (Tsuji et al., 2000). At the same time, anaerobic fermentation is increased, which accelerates lactic acid accumulation and free radical and glutamate production. Oxygen free radicals damage cell membrane lipids and block the synthesis and transport of genetic material, leading to cell apoptosis (Kaur and Ling, 2009); glutamate controls the opening of calcium channels, thereby activating lipase and endonuclease and ultimately inducing cell apoptosis (Volpe, 2001; Kaur and Ling, 2009).

Prelabor rupture of membranes can be caused by bacterial, viral, protozoan, chlamydia, mycoplasma, spirochetes, and other infections of the vaginal cavity (Yoo et al., 2017); this may lead to oligohydramnios and intra-amniotic cavity infection, which increase the risk of WMD (Lu et al., 2016). Following PROM, amniotic fluid level decreases, which puts greater pressure on the fetus. Fetal electronic monitoring frequently reveals late decelerations leading to FD (Roman et al., 2018). Intrauterine infections result in the production of various inflammatory factors such as tumor necrosis factor (TNF) and interleukins (ILs) that damage astrocytes, oligodendrocytes, and axons of the brain’s white matter (Berger et al., 2012). At the same time, inflammatory mediators can activate the body’s heat-producing center and cause fever, leading to a rise in fetal body temperature, increased oxygen and energy consumption in neurons, peroxide production, and oxygen free radical production, which further aggravate brain damage (Zhang et al., 2017).

We also found that prenatal administration of CS reduced the risk of neonatal WMD, which is consistent with previous findings. There are at least three possible reasons for this observed association. CS suppress immune responses related to infection or injury and thereby exert a protective effect on the neonatal nervous system as a result of increased permeability of the blood–brain barrier (Zammit et al., 2015). Alternatively, CS can promote fetal lung maturation, effectively preventing the occurrence of neonatal respiratory distress syndrome and reducing the risk of neonatal brain injury caused by hypoxia (Stoye et al., 2020). In addition, CS increase intracellular calcium availability in the cardiovascular system, leading to increased contractility of catecholamines in vascular smooth muscle cells (Wehling, 1997). CS also sensitize the cardiovascular system to endogenous and exogenous catechol by increasing the expression of adrenergic, dopaminergic, and angiotensin II receptors (Reynolds and Hanna, 1994; Ng et al., 2001, 2004; Seri and Noori, 2005; Seri, 2006). Consequently, another important effect of CS is blood pressure stabilization shortly after birth and prevention of hypotension that leads to a decrease of cerebral bleeding and WMD.

We based our risk prediction nomogram on prenatal and perinatal conditions of previous patients. We found that the incidence of WMD can be reduced by prolonging GA, actively preventing and controlling infection, and preventing intrauterine hypoxia. If preterm birth is inevitable, prenatal administration of CS can effectively reduce the risk of brain WMD (Scott and Rose, 2018). According to our model, the risk of neonatal WMD was assessed immediately after birth to distinguish high-risk and low-risk populations. After birth, resuscitation should be undertaken when necessary in high-risk neonates to prevent cerebral blood flow fluctuations and maintain normal cerebral perfusion pressure (Verhagen et al., 2009). If mechanical ventilation is required, blood gas analysis should be regularly performed to prevent hypocapnia (Wiswell et al., 1996; Sinha et al., 1997). This model could be used to decide in which infant ultrasound is recommended to confirm WMD. Regular follow-up should also be conducted after discharge in order to assess motor development and identify any abnormalities so that early intervention can be implemented to reduce the severity of disability by timely treatment/physiotherapy.

This study had some limitations. We used an internal validation method for our model, but external validation would provide greater rigor for data from other regions or hospitals. Additionally, our study had a retrospective design, and our ward is a follow-up treatment center for high-risk children in the province; moreover, most of the children who are admitted are low BW and early premature infants, so there was selection bias in the enrollment of patients in our cohort.

## Conclusion

We developed a nomogram and web-based calculator that showed good accuracy in predicting WMD risk in newborns. This model can be used to select infants at risk for WMD and perform cranial ultrasound in these for confirmation while avoiding excessive interventions for low-risk neonates, which can ensure better outcomes for WMD through optimized resource allocation and precision medicine.

## Data Availability Statement

The original contributions presented in the study are included in the article/Supplementary Material, further inquiries can be directed to the corresponding author/s.

## Ethics Statement

The studies involving human participants were reviewed and approved by Ethics Committee of The First Affiliated Hospital of Zhengzhou University (ID: 2020-KY-258). Written informed consent to participate in this study was provided by the participants’ legal guardian/next of kin.

## Author Contributions

QZ and CL conceived and designed the study. ML, MSo, MW, and MSh collected and sorted out the data. WC was responsible for drafting the manuscript. WD and JG was responsible for the production of part of the article diagram. The final version of the manuscript is calibrated and approved by all authors.

## Conflict of Interest

The authors declare that the research was conducted in the absence of any commercial or financial relationships that could be construed as a potential conflict of interest.

## Footnotes

1 https://www.r-project.org/

2 https://www.shinyapps.io/

3 https://caowenjun.shinyapps.io/dynnomapp/
