# Supplementary material for: Development and Validation of a Dynamic Nomogram to Predict the Risk of Neonatal White Matter Damage
Source: Front Hum Neurosci. 2021 Feb 23;14:584236. doi: 10.3389/fnhum.2020.584236 (PMC7940363; doi:10.3389/fnhum.2020.584236)
Supplement: Supplementary file 3 [file Table_3.DOCX]

Table 1: Baseline characteristics of cohort

|  | Total | Traning cohort | Validation cohort | P/Z |
| --- | --- | --- | --- | --- |
| WMD(%) | 157(9.06) | 114(9.38) | 43(8.32) | 0.483 |
| Birth characteristic |  |  |  |  |
| Male (%) | 923（53.26） | 667(54.85) | 256(49.52) | 0.042 |
| Cesarean delivery (%) | 1163（67.11） | 810(66.61) | 353(68.28) | 0.499 |
| Multiple pregnancy (%) | 240（13.85） | 182(14.97) | 58(11.22) | 0.039 |
| GA (weeks) (median [IQR]) | 36.86(34.29,39.14) | 36.86(34.29,39.14) | 36.86（34.43,39.29） | 0.320 |
| BW (g) (median [IQR]) | 2700(2050,3300) | 2700(2000,3300) | 2750(2100,3350) | 0.313 |
| Apgar score, 1 min (median [IQR]) | 9(8,10) | 9(8,10) | 10(8,10) | 0.532 |
| Apgar score, 5 min (median [IQR]) | 10(9,10) | 10(9,10) | 10(10,10) | 0.464 |
| Abnormal umbilical cord (%) | 465（26.83） | 324(26.64) | 141(27.27) | 0.787 |
| Placental abnormality (%) | 304（17.54） | 210(17.27) | 94(18.18) | 0.648 |
| Amniotic fluid anomaly (%) | 508（29.31） | 350(28.78) | 158(30.56) | 0.457 |
| Maternal characteristics |  |  |  |  |
| Maternal age (years) (median [IQR]) | 30(27,33) | 30(28,33) | 29(27,32) | 0.034 |
| GDM (%) | 300（17.31） | 217(17.85) | 83(16.05) | 0.367 |
| Hypothyroidism with pregnancy (%) | 94（5.42） | 66(5.43) | 28(5.42) | 0.992 |
| Hypertension (%) | 295（17.02） | 207(17.02) | 88(17.02) | 0.999 |
| ICP (%) | 27（1.56） | 17(1.40) | 10(1.93) | 0.410 |
| Cardiac dysfunction (%) | 13（0.75） | 5(0.41) | 8(1.55) | 0.027 |
| History of abnormal pregnancy (%) | 178（10.27） | 133(10.94) | 45(8.70) | 0.161 |
| Maternal disease (%) | 326（18.81） | 234(19.24) | 92(17.79) | 0.48 |
| Pregnancy-related factors |  |  |  |  |
| Embryo transfer (%) | 324（18.70） | 243(19.98) | 81(15.67) | 0.035 |
| Fetal distress (%) | 203（11.71） | 140(11.51) | 63(12.19) | 0.69 |
| IUGR (%) | 67（3.87） | 44(3.62) | 23(4.45) | 0.412 |
| Use of CS (%) | 193（11.14） | 132(10.86) | 61(11.80) | 0.568 |
| Pregnancy treatment (%) | 247（14.25） | 171(14.06) | 76(14.70) | 0.728 |
| PROM (%) | 416（24.00） | 302(24.84) | 114(22.05) | 0.214 |
